# Parallel Development of Chromatin Patterns, Neuron Morphology, and Connections: Potential for Disruption in Autism

**DOI:** 10.3389/fnana.2018.00070

**Published:** 2018-08-17

**Authors:** Miguel Á García-Cabezas, Helen Barbas, Basilis Zikopoulos

**Affiliations:** ^1^Neural Systems Laboratory, Department of Health Sciences, Boston University, Boston, MA, United States; ^2^Graduate Program in Neuroscience, Boston University, Boston, MA, United States; ^3^Human Systems Neuroscience Laboratory, Department of Health Sciences, Boston University, Boston, MA, United States

**Keywords:** projection neuron, limbic cortex, histone modification, epigenetics, neural pathways, development, autism, open chromatin

## Abstract

The phenotype of neurons and their connections depend on complex genetic and epigenetic processes that regulate the expression of genes in the nucleus during development and throughout life. Here we examined the distribution of nuclear chromatin patters in relation to the epigenetic landscape, phenotype and connections of neurons with a focus on the primate cerebral cortex. We show that nuclear patterns of chromatin in cortical neurons are related to neuron size and cortical connections. Moreover, we point to evidence that reveals an orderly sequence of events during development, linking chromatin and gene expression patterns, neuron morphology, function, and connections across cortical areas and layers. Based on this synthesis, we posit that systematic studies of changes in chromatin patterns and epigenetic marks across cortical areas will provide novel insights on the development and evolution of cortical networks, and their disruption in connectivity disorders of developmental origin, like autism. Achieving this requires embedding and interpreting genetic, transcriptional, and epigenetic studies within a framework that takes into consideration distinct types of neurons, local circuit interactions, and interareal pathways. These features vary systematically across cortical areas in parallel with laminar structure and are differentially affected in disorders. Finally, based on evidence that autism-associated genetic polymorphisms are especially prominent in excitatory neurons and connectivity disruption affects mostly limbic cortices, we employ this systematic approach to propose novel, targeted studies of projection neurons in limbic areas to elucidate the emergence and time-course of developmental disruptions in autism.

## Introduction

Neurons are highly specialized cells that perform a wide variety of functions. Functional specialization depends on morphological, biochemical and physiological features of neurons. Accordingly, neurons can be classified based on a multitude of features including: (1) the number of dendritic trunks, which distinguish unipolar, bipolar, and multipolar neurons; (2) the length of the axon, which separates long axon (projection) from short axon (local circuit) neurons; (3) the specialization of the dendrites, which distinguishes spiny and non-spiny neurons; and (4) the neurotransmitter, which differentiates excitatory (glutamatergic in the central nervous system, cholinergic in the peripheral nervous system), inhibitory (GABAergic or glycinergic), or modulatory (e.g., dopaminergic) neurons. The combination of morphological and biochemical features exhibited by a given neuron underlies its function. For instance, projection neurons in the cerebral cortex are long axon-spiny-excitatory neurons and most are pyramidal but some are fusiform (von Economo cells). In contrast, the recipients of thalamic projections in cortical layer IV are local circuit-spiny-excitatory neurons with stellate cell body, but some are small pyramids. Inhibitory neurons across layers in the cerebral cortex are local circuit neurons with non-spiny or sparsely spiny dendrites and their body shape can be stellate, fusiform or triangular (Ramón y Cajal, 1899/2002; Mitra, 1955; Ramon-Moliner, 1962, 1967; Valverde, 1971; Lund, 1973; Valverde, 1978; Lund et al., 1981; Peters and Jones, 1984; Jones, 1985; Peters et al., 1991; DeFelipe, 2002; Valverde, 2002; Migliore and Shepherd, 2005; Watson et al., 2006).

The development and maintenance of structural and functional features of cells depend on the expression of genes in the nucleus. The pattern of gene expression is characteristic of each cell class as shown for glial cells and distinct subclasses of excitatory and inhibitory neurons in the cerebral cortex (Zeisel et al., 2015; Lake et al., 2016; Tasic et al., 2016). Genes whose transcription mediates cellular function are easily accessible in an “open euchromatin” mode. In contrast, genes that are not transcribed in a given cell are compacted into heterochromatin aggregates (reviewed in Bouteille et al., 1974; Fedorova and Zink, 2008; Jost et al., 2012; Deneris and Hobert, 2014; Tessarz and Kouzarides, 2014). Accordingly, differences in gene accessibility condition different programs of gene expression across cells and tissues (Qu and Fang, 2013; Andersson et al., 2014; Roadmap Epigenomics et al., 2015; Schultz et al., 2015). The nuclear distribution of euchromatin and heterochromatin in neurons can be seen readily in sections stained with classical cellular stains, such as Nissl, or in sections stained with heavy metals (Merchán et al., 1933/2016; Ramón y Cajal, 1933; Lafarga et al., 1991; Peters et al., 1991; García-Cabezas et al., 2016). Surprisingly, there are few descriptions of nuclear chromatin patterns and their relation with the morphological and biochemical features of neurons. Recent reviews on nuclear and nucleolar architecture of neurons address multiple aspects of molecular organization of chromatin but do not comment on differences across neuron classes or brain regions (e.g., Takizawa and Meshorer, 2008; Hetman and Pietrzak, 2012; Moccia and Martin, 2018) with the exception of photoreceptors (Alexander and Lomvardas, 2014). Epigenetic studies describe different chromatin signatures across glial cell types and neuron subclasses, but they do not address their relation with nuclear structure (e.g., Fullard et al., 2017; Lake et al., 2017; Luo et al., 2017), with the exception once again of photoreceptors (Hughes et al., 2017). Moreover, little is known about the relation between neuronal patterns of chromatin organization and neural connections.

Here we review the patterns of nuclear chromatin in relation with morphological, biochemical, and connectional features of neurons with a focus on the cerebral cortex in the rodent and primate brain. We conclude that the nuclear pattern of chromatin and the epigenetic landscape in neurons across areas in the adult cortex is related to cortical connections, a relationship that can be traced to development. Importantly, the sequence of events in the development of chromatin patterns, gene expression, neuron morphology, and connections across cortical areas and layers are likely disrupted in disorders of neurodevelopmental connectivity like autism spectrum disorders (ASD). Accordingly, the epigenetic marks of specific cortical neurons in individuals with ASD will reflect the developmental disruption of pathways of these disorders.

## Nuclear Patterns of Chromatin Distinguish Neurons by Cell Body Size

The patterns of nuclear chromatin of nerve cells were first described by Santiago Ramón y Cajal in Nissl stained sections. Cajal showed that the disposition of nuclear chromatin was not related to the function of the neuron but to the size of its nucleus and differentiation of the cytoplasm: the denser the Nissl substance (composed mostly of ribosomes) in the cytoplasm, the more concentrated and simplified was the nuclear chromatin (Ramón y Cajal, 1896; Ramón y Cajal, 1899/2002). Cajal also showed that the number of argyrophilic spheres embedded in the interstitial matter of the nucleoli of human cortical neurons was related to the size of the cell. The number of argyrophilic spheres ranged from about 5 in ‘short axon neurons’ (which are small) to about 30 in large pyramidal neurons (Ramón y Cajal, 1909/1952, 1910). Argyrophilic spheres likely correspond to the ultrastructure of fibrillary centers in *pars fibrosa* of neuronal nucleoli (Peters et al., 1991; Lafarga et al., 2009, 2017) that contain the nucleolar organizer regions of DNA (Manuelidis, 1984a,b) that encode ribosomal RNA (Busch and Smetana, 1970). Accordingly, more and larger fibrillary centers are related to higher rates of ribosome production (Sirri et al., 2008).

**FIGURE 1:**
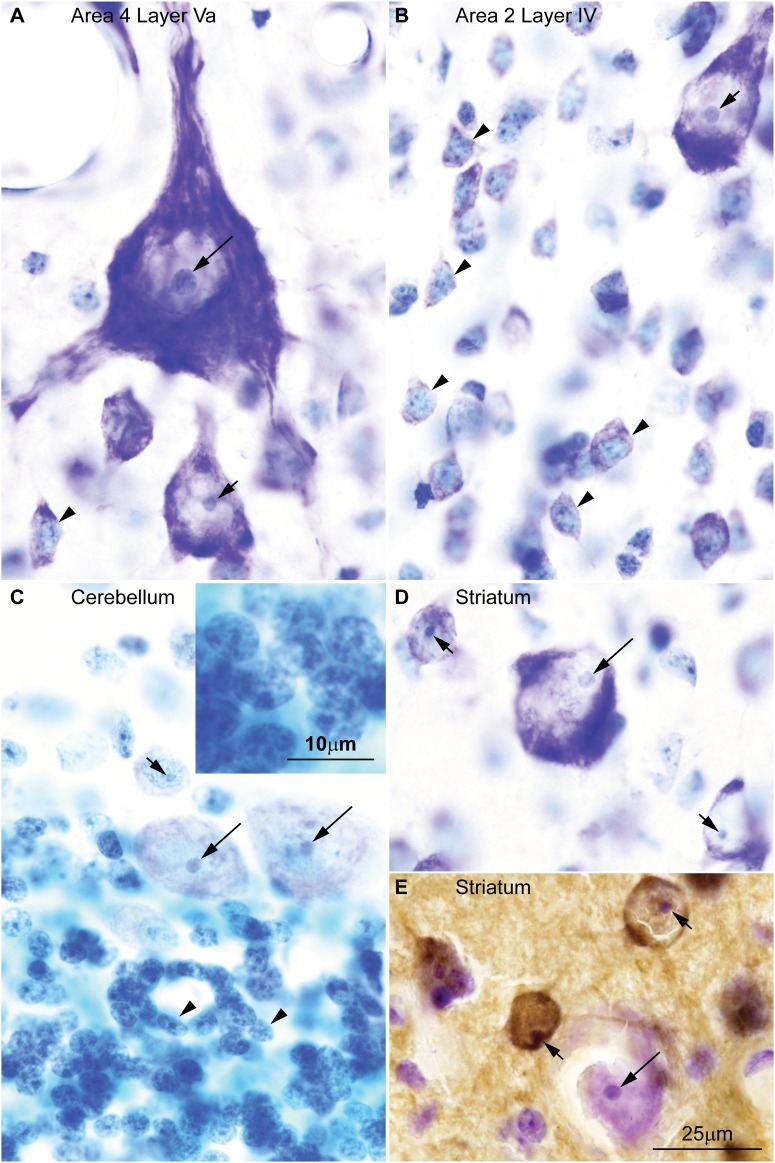
Chromatin patterns shown by Nissl are not related to neurotransmitter expression across the central nervous system of the rhesus macaque. **(A)** Micrograph of layer Va in area 4 of the cerebral cortex shows a Betz cell, the largest neuron type in the cortex, with large distinct nucleolus (long arrow) in an “empty”-looking nucleus; large pyramids are glutamatergic projection neurons and have few heterochromatin grains around the nucleolus (short arrow); small non-pyramidal neurons, likely short axon neurons, have a small nucleolus surrounded by clumps of chromatin and their euchromatin is lightly stained (arrowhead). **(B)** In layer IV of cortical area 2, small neurons (arrowheads) have several grains and clumps of heterochromatin and their euchromatin is lightly stained; pyramidal cells of adjacent layer IIIb show larger nucleoli (short arrow) with fewer heterochromatin grains in an “empty”-looking nucleus. **(C)** Purkinje cells in the cerebellum are GABAergic and have large distinct nucleolus (long arrows) within the “empty”-looking nucleus; smaller short axon neurons in layer I of the cerebellum (above Purkinje cells) have smaller nucleolus surrounded by several grains of heterochromatin (short arrow); granule cells, which are the smallest neurons in the central nervous system, are glutamatergic and have multiple grains and clumps of heterochromatin forming a continuous net in the nucleus (arrowheads; see inset on the upper left corner). **(D)** Large interneurons in the striatum are cholinergic and, like large cortical pyramids and Purkinje cells, have a large nucleolus (long arrow) in an “empty”-looking nucleus; medium size spiny neurons are GABAergic and have smaller nucleolus (short arrows) with some few heterochromatin grains. **(E)** DARPP-32 staining labels medium size spiny neurons (brown, short arrows); cholinergic interneurons are not labeled by DARPP-32 (long arrow). Calibration bar in **(E)** applies to **(A–E)**. This figure is based on reexamination of material from two earlier papers: **(A–D)** García-Cabezas et al. (2016) **(E)** Barbas et al. (1993).

We recently developed an algorithm that is highly effective in training experienced or untrained individuals alike to identify and distinguish neurons and glial cells stained for Nissl with high inter-observer reliability (García-Cabezas et al., 2016). According to this algorithm, large neurons in the cerebral cortex, which are glutamatergic projection neurons, have a large nucleolus and virtually lack heterochromatin. In contrast, small neurons in the cortex have more heterochromatin granules that may surround entirely a smaller nucleolus (**Figures 1A,B**). Large neurons in other parts of the brain, like Purkinje cells in the cerebellum which are GABAergic (**Figure 1C**) or cholinergic interneurons in the striatum (**Figures 1D,E**), also have a large nucleolus with scant heterochromatin. The largest amount of heterochromatin and the smallest nucleolus is found in the nuclei of the smallest neurons, like glutamatergic granule cells of the cerebellum (**Figure 1C**).

A quick survey of neuron types in the central nervous system shows that the pattern of nuclear chromatin is not related to the biochemical nature of the neuron or the presence of spines in their dendritic tree (**Table 1**). For instance, projection neurons in the olfactory bulb and in the cerebral cortex are excitatory and show comparable nuclear chromatin patterns, but the former are non-spiny and the latter are spiny (Peters and Jones, 1984; Ramón y Cajal, 1904/2002). In the striatum there are cholinergic local circuit neurons and projection neurons that show nuclear chromatin patterns comparable to glutamatergic projection neurons in the cerebral cortex. However, cholinergic interneurons have smooth dendrites while striatal projection neurons are GABAergic and spiny (Fox et al., 1971; DiFiglia et al., 1976; Poirier et al., 1977). This brief survey supports classical observations that chromatin patterns are related to the size of the neuron, but not to the presence of dendritic spines or to a specific neurotransmitter.

**Table 1:** Morphological features, neurotransmitter, and chromatin patterns of central neurons.

	Large nucleolus scant heterochromatin			Small nucleolus abundant heterochromatin		
	Spiny	Long axon	Neuro transmitter	Spiny	Long axon	Neuro transmitter
*Cortex* Projection neuron	+	+	Glutamate			
*Cortex* Inhibitory interneuron				−	−	GABA
*Cortex* Excitatory interneuron				+	−	Glutamate
*Olfactory bulb* Projection neuron (Mitral/tufted cell)	−	+	Glutamate			
*Olfactory bulb* Interneuron (granule cell)				+	−	GABA
*Striatum* Projection neuron	+	+ (medium-range)	GABA			
*Striatum* Cholinergic interneuron	−	−	Acetylcholine			
*Cerebellum* Projection neuron (Purkinje cell)	+	+ (medium-range)	GABA			
*Cerebellum* Interneuron (granule cell)				−	−	Glutamate

## Patterns of Chromatin Distribution Distinguish Projection and Local Circuit Neurons in the Cerebral Cortex

Classical studies have indicated that the size and complexity of dendrites and axons of pyramidal neurons in the cerebral cortex increase from rodents to humans (Ramón y Cajal, 1904/2002; Bok, 1959). Modern studies have provided some evidence to support these evolutionary trends (Valverde, 1986, 2002; Elston et al., 2001; Gilman et al., 2017), and have additionally associated increases in neuron number and morphological complexity of dendritic and axonal processes with the expansion of the cortex, growth of connections, and increase in the distance between interconnected areas (Schenker et al., 2005; Herculano-Houzel, 2009). A major contribution of modern studies is the use of precise morphometric measures to systematically show increased variation in pyramidal neuron structure across cortical areas in primates (Elston, 2003) compared to rodents (Benavides-Piccione et al., 2006).

Comparison of local circuit versus projection neurons in the cortex shows that the length of the axon, the size of the nucleus, and the size of the nucleolus increase with the size of neuron body, while the amount of heterochromatin decreases. Thus, the large neurons in layers IIIb and Va of primate species have large nuclei, which appear “empty” with Nissl stain and have a well-defined nucleolus with small heterochromatin grains attached to it (**Figures 1A,B**). These large neurons of the cortex are projection neurons; they have extensive and complex dendritic trees and long axons. The largest neurons in the human and rhesus macaque cortex are the Betz cells in the primary motor cortex, which have long axons, like other layer V neurons (Scheibel and Scheibel, 1978). Betz cells have the largest nucleus and nucleolus with almost imperceptible grains of heterochromatin (**Figure 1A**, long arrow). In contrast to long-axon neurons, local-circuit neurons of the cerebral cortex, like small cells of layer IV, have short axons, short dendritic trees, small nuclei with smaller nucleoli, more heterochromatin clumps, and lightly stained euchromatin (**Figure 1B**, arrowheads). These nuclear features can be used to distinguish local circuit neurons in layer IV of different cortical areas (García-Cabezas and Barbas, 2014).

The maintenance of long axons likely increases the production of ribosomes for the synthesis of more proteins, and one large, well-defined and compacted nucleolus might be the most effective way to achieve this (Hetman and Pietrzak, 2012). For instance, motor neurons in the cervical and lumbosacral segments of the spinal cord have larger nucleoli than motor neurons in thoracic segments, whose axons are shorter (Koya and Friede, 1969). However, the length of the axon is likely not the only factor that influences the size of the cell body, the chromatin pattern, and the size of the nucleolus of neurons. Cajal addressed this question and concluded that the size of the neuron body is approximately proportional to the number and thickness of the branches of the axon, and likely to the amount of synaptic contacts of the axon (Ramón y Cajal, 1909/1952). Accordingly, the maintenance of long axons (as shown for spinal cord motor neurons) and/or axons with highly ramified telodendria and more synaptic contacts (as suggested by Cajal) would require more protein synthesis and larger nucleoli.

**FIGURE 2:**
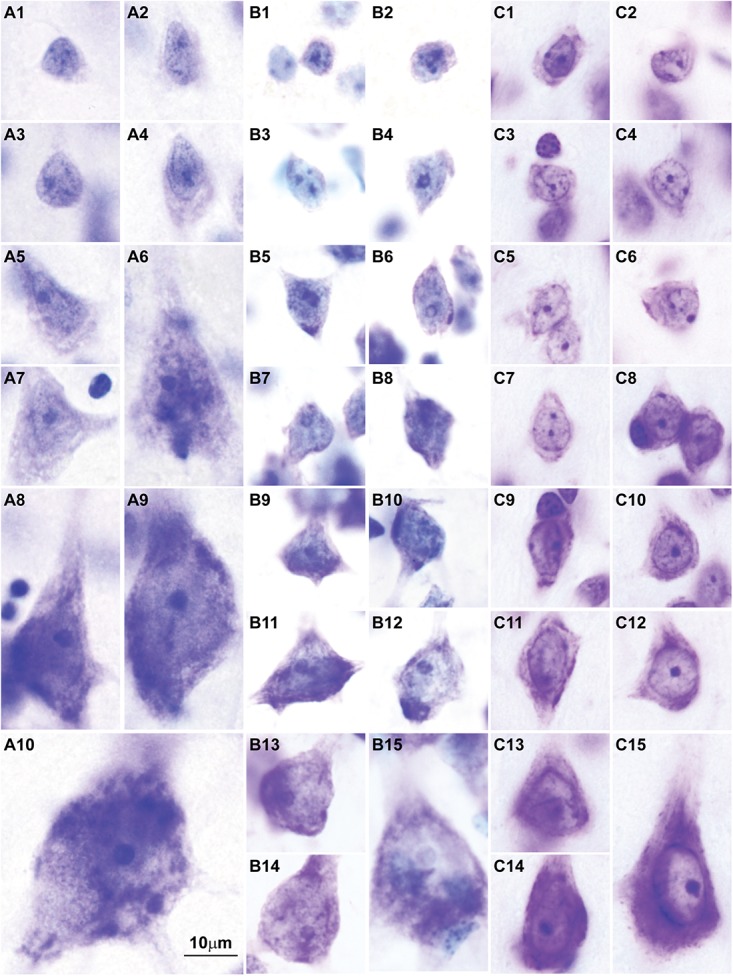
Nuclear chromatin patterns shown by Nissl staining are related to neuron size in the cerebral cortex. High magnification photomicrographs of neurons in the human **(A1–A10)**, in the rhesus macaque **(B1–B15)**, and in the rat **(C1–C15)** show progressively fewer heterochromatin grains and larger nucleoli as neuron body size increases. Smaller neurons show comparable sizes and heterochromatin distribution across species (**A1–A4**, human; **B1–B4**, rhesus macaque; **C1–C4**, rat). Large neurons are larger in the human **(A9,A10)** than in the rhesus macaque **(B13–B15)** and in the rat **(C15)**. Calibration bar in **(A10)** applies to all panels. **(A1–A10, B1–B15)** of this figure are a reexamination of material form an earlier paper (Zikopoulos et al., 2018); **(C1–C15)** are a reexamination of material form a gift of Dr. Alan Peters.

**FIGURE 3:**
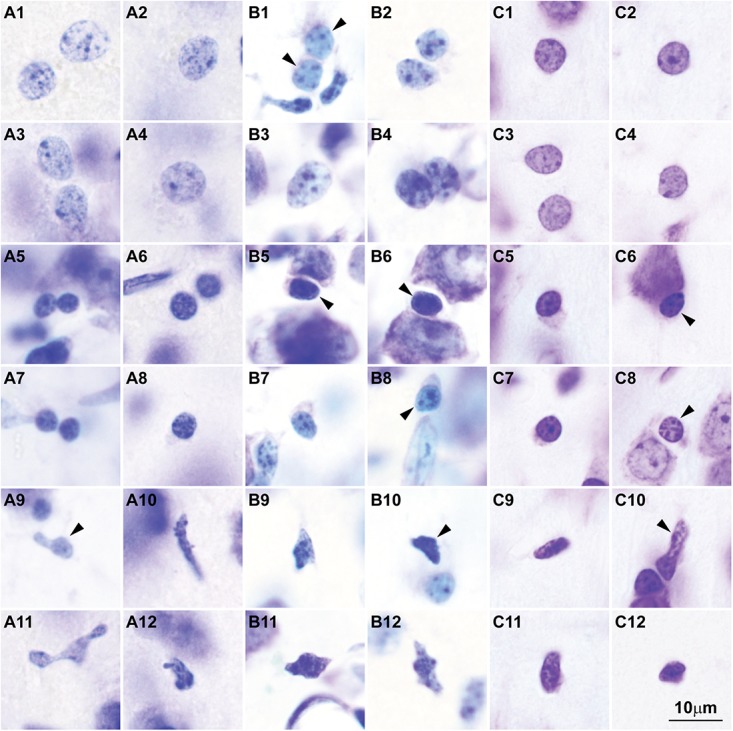
Nuclear chromatin patterns and nuclear sizes of glial cells in the cerebral cortex shown by Nissl are comparable across primate and non-primate species. **(A1–A12)** Photomicrographs of human astrocytes **(A1–A4)**, oligodendrocytes **(A5–A8)**, and microglial cells **(A9–A12)**. **(B1–B12)** Micrographs of rhesus macaque astrocytes **(B1–B4)**, oligodendrocytes **(B5–B8)**, and microglial cells **(B9–B12)**. **(C1–C12)** Micrographs of rat astrocytes **(C1–C4)**, oligodendrocytes **(C5–C8)**, and microglial cells **(C9–C12)**. Arrowheads in panels showing more than one glial cell type point to astrocytes **(B1)**, oligodendrocytes **(B5,B6,B8, C6, C8)**, and microglial cells **(A9, B10, C10)**. Calibration bar in **(C12)** applies to all panels. **(A1–A12)** and **(B1–B12)** of this figure are a reexamination of material form an earlier paper (Zikopoulos et al., 2018); **(C1–C12)** are a reexamination of material form a gift of Dr. Alan Peters.

Here we surveyed and compared neurons in Nissl stained sections of cortical areas of humans, rhesus macaques, and rats systematically for the first time. Our analysis showed that chromatin patterns in cortical neurons are graded along body size, with progressively fewer heterochromatin grains and clumps in larger neurons (**Figure 2**). Interestingly, small neurons do not differ in size and heterochromatin distribution pattern across these species. By contrast, large neurons in primates are considerably larger than in rats, a feature that likely reflects the increase in the length of the axon of projection neurons in larger brains. In contrast with neurons, the nuclei of glial cell types in the cortex show comparable sizes and chromatin patterns across species (**Figure 3**).

## Projection Neurons in the Cerebral Cortex Have More Accessible Chromatin Regions

The findings of recent studies of epigenetic states that measure chromatin accessibility in thousands of single cells can be best understood in the light of chromatin patterns of neurons and glial cell types. These studies show higher variability of open chromatin regions in neurons across cortical areas (Fullard et al., 2017, 2018; Lake et al., 2017), as predicted by varied patterns of nuclear chromatin (**Figures 1**, **2**). Cerebellar granule cells stand apart from cortical excitatory and inhibitory neurons (Lake et al., 2017), as predicted by their unique nuclear pattern (**Figure 1C**). In contrast, glial cell types seem to have comparable open chromatin regions across cortical and subcortical regions (Lake et al., 2017; Fullard et al., 2018), as predicted by their constant patterns of nuclear chromatin (**Figure 3**).

Studies of epigenetic states also show more open and accessible chromatin in large than in small neurons and glial cells. For instance, in human cortical neurons open chromatin regions are more extensive than in glial cells (Fullard et al., 2018), which may reflect the larger amount of euchromatin in large pyramids (**Figures 1**, **2**). In line with this, excitatory neurons in the mouse and in the human frontal cortex have more hypo-methylated DNA regions than inhibitory interneurons (Mo et al., 2015; Kozlenkov et al., 2016). Large neuron nuclei across the central nervous system of rats and pigs also have more decondensed chromatin and increased turnover rate of chromatin modification (Yu et al., 2015). A major characteristic of large projection neurons in the cerebral cortex is that their euchromatin is not stained with the Nissl technique and the nucleus looks “empty”. In contrast, in small neurons the euchromatin has a light blue hue (García-Cabezas et al., 2016; **Figures 1A,B**). Different staining patterns with basic dyes, like those used in Nissl, suggest different chemical properties of euchromatin across neurons that could be attributed to a higher degree of decondensation of chromatin. The most decondensed chromatin is composed of 10 nm basic chromatin fibers in which histone cores and linker-histones are more exposed compared to 30 nm fibers (Solari, 1974). Histones are highly alkaline proteins (Akinrimisi et al., 1965). The lack of staining with basic dyes in the nuclei of large neurons suggests that their euchromatin is enriched in 10 nm chromatin fibers, the ultrastructural counterpart of open chromatin regions.

We can conclude that large projection neurons in the cortex have more open chromatin regions than small neurons. Importantly, open chromatin regions are enriched in distal regulatory elements like enhancers and promoters in human cortical neurons compared to glial cells (Fullard et al., 2017). Are these epigenetic differences between neuron and glial cell types and between classes of large and small neurons related to the transcription of more genes or of more transcripts of genes? Transcription studies show that neurons in the cerebral cortex of mice, in particular excitatory cortical neurons, contain more RNA and express more genes than glial and vascular cells (Zeisel et al., 2015; Tasic et al., 2016). In the human cortex more genes are expressed in excitatory than in inhibitory neurons (Lake et al., 2016). However, differences between excitatory and inhibitory neurons of the cortex are smaller than between neurons and glial cells, probably because the expression profiles of small excitatory interneurons are mixed with the profiles of large projection neurons. Further studies are needed to address the magnitude of differences in transcription among cortical neuron types.

## Major Output Layers of Cortical Areas Have Larger Neurons With Open Chromatin

Analyses of nuclear architecture and chromatin patterns of neurons, using sections processed for cellular stains, provide the framework to place single-cell studies of transcriptional and epigenetic states within the context of the organization of brain systems and networks. The gradual changes in chromatin patterns and soma size of neurons parallel and may underlie gradual architectonic changes in the structure of cortical areas, which varies systematically and follows a similar pattern across mammals, including humans. Limbic areas at the foot of every cortical lobe and system either lack (agranular), or have a rudimentary layer IV (dysgranular) and overall have poor laminar differentiation. In contrast, eulaminate areas have well developed layer IV and better laminar distinction (reviewed in Sanides, 1970; Barbas, 2015; Barbas and García-Cabezas, 2015). The laminar structure of the cortex is associated with the laminar pattern of connections between areas, formulated by the Structural Model for Connections (Barbas and Rempel-Clower, 1997). According to this model, connections between areas are biased toward a feedforward or feedback pattern, depending on the (dis)similarities of their architecture. The bigger the architectonic differences the bigger the bias. For example, agranular and dysgranular (limbic) areas send mainly feedback projections to eulaminate areas. These connections originate mainly from the deep layers and terminate mostly in the superficial layers of the target area. Projections in the opposite direction, from eulaminate to limbic areas, are feedforward: they originate mostly from the superficial layers and terminate in the middle/deep layers. Connections between areas with similar architecture are columnar, originating in most layers and terminating in all layers (reviewed in Barbas, 2015).

**FIGURE 4:**
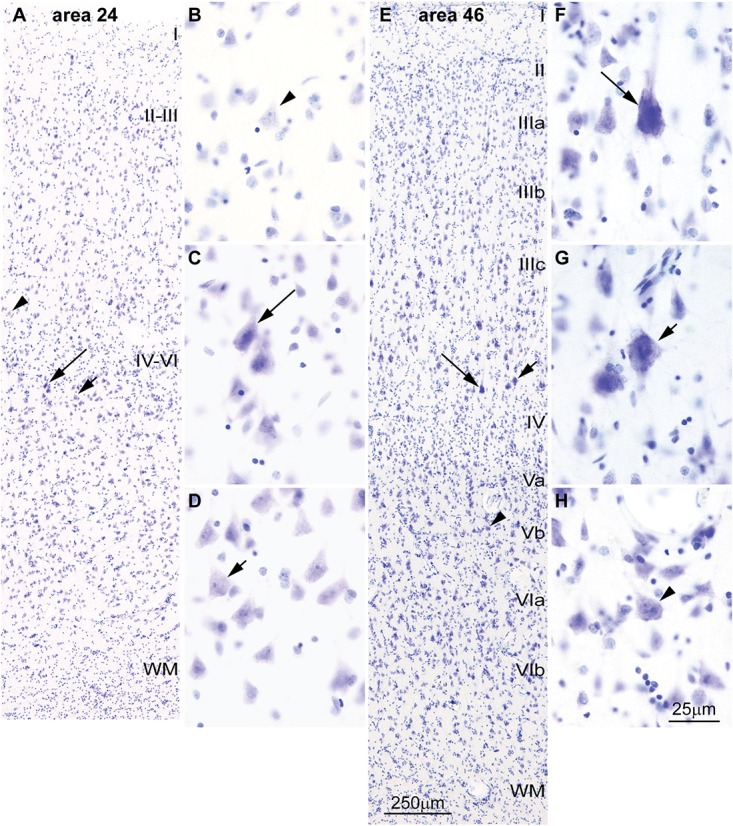
Differences in the laminar distribution of large pyramids across areas in the human cerebral cortex. **(A–D)** The largest pyramidal neurons in limbic area 24 in the subgenual cingulate cortex are in layer V (arrows); pyramids in layer III are comparatively smaller (arrowhead). **(E–H)** The largest pyramidal neurons in eulaminate area 46 in the dorsolateral prefrontal cortex are in layer III (arrows), while pyramids in layers V area smaller (arrowhead). Pyramids in layer V of limbic area 24 **(C,D)** are overall smaller than pyramids in layer III of eulaminate area 46 **(F,G)**. WM, white matter. Roman numerals indicate cortical layers. Calibration bar in **(E)** applies to **(A,E)**. Calibration bar in **(H)** applies to **(B–D,F–H)**. (This figure is a reexamination of material from an earlier paper; Zikopoulos et al., 2018).

**FIGURE 5:**
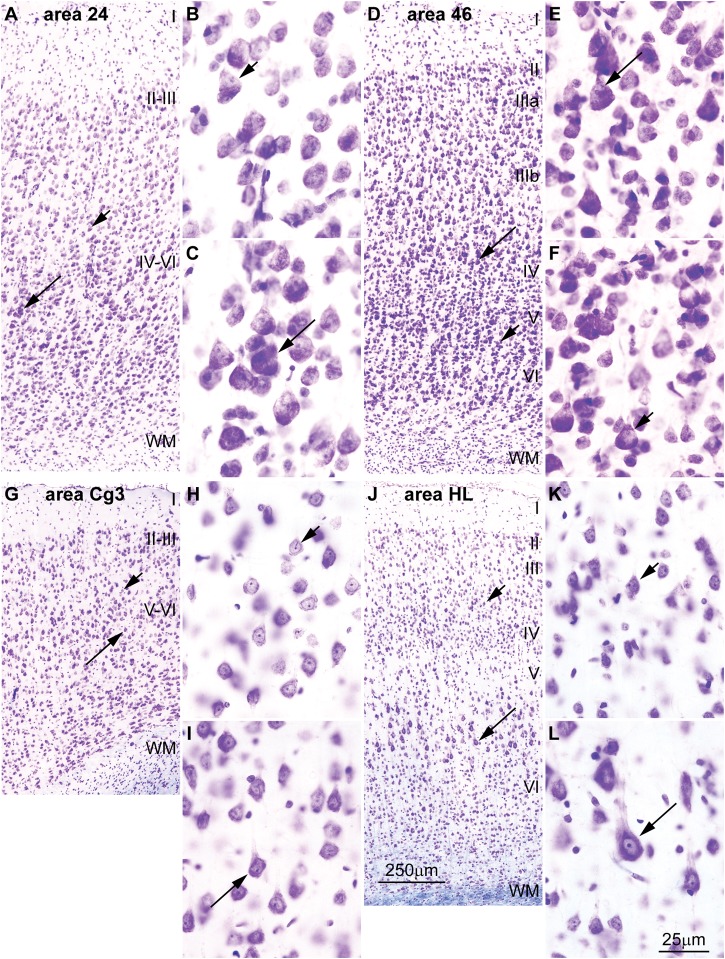
Differences in the distribution of large pyramids in the rhesus macaque and rat cerebral cortex. **(A–C)** The largest pyramidal neurons in limbic area 24 in the subgenual cingulate cortex of the rhesus macaque are in layer V (long arrow), while pyramids in layer III are comparatively smaller (short arrow). **(D–F)** Pyramidal neurons in eulaminate area 46 in the dorsolateral prefrontal cortex of the rhesus macaque are slightly larger in layer III (long arrow) than in layer V (short arrow). **(G–I)** The largest pyramidal neurons in agranular area Cg3 of the rat cortex are in layer V (long arrow), while pyramids in layer III are smaller (short arrow). **(J–L)** The largest pyramidal neurons in granular area HL of the rat cortex are also found in layer V (long arrow) and pyramids in layer III are smaller (short arrow). Cg3, cingulate area 3; HL, hind limb area; WM, white matter. Roman numerals indicate cortical layers. Calibration bar in **(J)** applies to **(A,D,G,J)**. Calibration bar in **(L)** applies to **(B,C,E,F,H,I,K,L)**. **(A–F)** of this figure are a reexamination of material from an earlier paper (Zikopoulos et al., 2018); **(G–L)** are a reexamination of material form a gift of Dr. Alan Peters.

Cortical areas have relatively more neurons in their primary output layers: Layer V for limbic cortices and layer III for eulaminate cortices (Barbas, 1986; Barbas and Rempel-Clower, 1997; Dombrowski et al., 2001; Zikopoulos et al., 2018). This pattern is also reflected in the size of pyramidal projection neurons. In the human cortex, neurons are larger in layer V for limbic areas (**Figures 4A–D**), while they are larger in layer III in eulaminate cortices (Sanides, 1970; Goulas et al., 2018; Zikopoulos et al., 2018; **Figures 4E–H**]. The differences in the size of cortical projection neurons across layers in limbic and eulaminate areas are less prominent in rhesus macaques compared to humans (**Figures 5A–F**). In rats, the largest pyramidal neurons across cortical areas are found in layer V (**Figures 5G–L**), as shown in classical (Krieg, 1946) and modern studies (Zilles, 1985; Goulas et al., 2018).

Recent studies also show larger variability in the dendritic structure of pyramidal neurons across cortical areas of primates compared to rodents. In a series of detailed studies, Elston and colleagues filled the cytoplasm and analyzed the basal dendrites of about 2,000 pyramidal neurons in layer III across 27 cortical areas from more than 15 macaques. The data from these studies (summarized in **Table 2**) show that pyramids in primary sensory areas (visual, somesthesic, and auditory), in the primary motor area, and in extrastriate areas of the occipital lobe have the smallest basal dendritic fields across cortical areas of the macaque. In contrast, pyramids in limbic areas in the cingulate cortex have the largest dendritic fields in the macaque cerebral cortex, ten times larger than in the primary visual area. We have suggested that dendrite complexity of pyramidal neurons decreases in parallel with progressive laminar elaboration from limbic to eulaminate cortices (Garcia-Cabezas et al., 2017). A quick comparison of the basal dendritic field area of pyramids in layer III with the structural type of the cortical area (Barbas and Rempel-Clower, 1997; Hilgetag et al., 2016) shows that, in general, the size of the basal dendritic field area of pyramids in layer III decreases in parallel with laminar elaboration (**Table 2**). Studies on the dendritic structure of pyramidal neurons in rodents are scarce and less systematic than in primates, but overall they show smaller basal dendritic trees than in macaques (e.g., Elston et al., 1999a) and smaller variation in basal dendritic trees of pyramidal neurons across cortical areas in rodents (Benavides-Piccione et al., 2006; Gilman et al., 2017). This is not surprising because variation in laminar structure of the cortex of rodents is less pronounced than in primates (Reep, 1984; Goulas et al., 2018). Data summarized in **Table 2** and our present observations suggest that larger variation of dendritic fields and neuron body size parallels laminar elaboration. The size of dendritic fields and neuron bodies of small cortical neurons is comparable across rats and primates, but primates have a set of larger pyramidal neurons with more open chromatin whose laminar distribution is related to architectonic differentiation. To sum up, congruent changes in the variability of laminar elaboration, neuron size, pyramidal dendritic complexity, and nuclear patterns of chromatin, reveal a fundamental organizational principle of the cerebral cortex.

**Table 2:** Systematic variation of basal dendritic field area of pyramidal neurons in layer III across the macaque cortex parallels laminar elaboration.

Cortical area	Basal dendritic field area (×10^4^μm^2^) ^∗^Average across cases	Structural type (Barbas and Rempel-Clower, 1997; Hilgetag et al., 2016)	Reference
V1i (primary visual area, interblobs)	2	8	Elston and Rosa, 1998a
V1b (primary visual area, between blobs)	2.69	8	Elston and Rosa, 1998a
V1 (primary visual area)	^∗^4.08	8	Elston and Rosa, 1997; Elston et al., 2005
V2 interstripe (secondary visual area, cytochrome oxidase poor interstripes)	4.23	7	Elston and Rosa, 1998a
V2 (secondary visual area)	^∗^4.43	7	Elston and Rosa, 1997; Elston et al., 2001
V2 thin stripe (secondary visual area, cytochrome oxidase rich stripes)	4.63	7	Elston and Rosa, 1998a
3b (primary somesthesic area 3b)	4.91	–	Elston and Rockland, 2002
A1 (primary auditory area)	5.05	–	Elston et al., 2010
V4 (occipital extrastriate visual area 4)	7.47	6	Elston and Rosa, 1998a
4 (primary motor area)	7.49	–	Elston and Rockland, 2002
5 (posterior parietal area 5)	8.06	–	Elston and Rockland, 2002
TEa (inferior temporal area TEa)	8.19	–	Elston et al., 2001
MT (middle temporal area)	8.38	6	Elston and Rosa, 1997
7a (posterior parietal area 7a)	8.47	4	Elston and Rosa, 1997
LIPv (ventral portion of the lateral intraparietal area)	8.81	5	Elston and Rosa, 1997
6 (Premotor area 6)	9.03	–	Elston and Rockland, 2002
7b (posterior parietal area 7b)	9.60	–	Elston and Rockland, 2002
TE (inferior temporal area TE)	9.70	–	Elston et al., 1999b
7m (posterior parietal area 7m)	^∗^10.9	–	Elston et al., 1999c; Elston, 2001
FEF (frontal eye field)	11.5	5	Elston and Rosa, 1998b
46 (dorsolateral prefrontal area 46)	^∗^11.5	4–5	Elston et al., 2011
STP (superior temporal polysensory area)	^∗^12	4	Elston et al., 1999b; Elston, 2001
13 (posterior orbitofrontal area 13)	^∗^12	2	Elston et al., 2011
TEO (inferior temporal area TEO)	12.30	6	Elston and Rosa, 1998a
11 (orbitofrontal area 11)	12.30	3	Elston, 2000
9 (dorsolateral prefrontal area 9)	^∗^12.35	4	Elston et al., 2011
IT (inferior temporal cortex)	^∗^12.70	–	Elston et al., 2005
10 (frontopolar area 10)	^∗^12.85	4	Elston, 2000; Elston et al., 2001, 2011
12 (dorsolateral prefrontal area 12)	^∗^12.90	3–4	Elston, 2000; Elston et al., 2011
23 (posterior cingulate area 23)	^∗^13.30	–	Elston et al., 2005
24 (anterior cingulate area 24)	^∗^18.40	1	Elston et al., 2005

We have recently shown in primates that eulaminate lateral prefrontal areas have relatively more long-range connections than limbic areas in the anterior cingulate cortex (Zikopoulos et al., 2018). In line with this observation, the major output pyramidal projection neurons in layer III of lateral prefrontal areas are larger and have less heterochromatin than the major output neurons in layer V of anterior cingulate (limbic) areas (**Figure 4**). A quick survey of the human cortex supports this observation for most areas and systems and allows formulation of a general principle: Layer III projection neurons in eulaminate cortices are among the largest in the cortex with the most open chromatin (euchromatin). These neurons connect the most distant cortical areas through long-range pathways. This applies both to long-range ipsilateral cortico-cortical connections (Zikopoulos et al., 2018), as well as contralateral projections, most of which originate in pyramidal neurons in layer III (Barbas et al., 2005). This principle also applies to the primary motor cortex, where pyramids in layer III are larger than in layer V with the exception of Betz cells (Ramón Y Cajal, 1899/1988). Systematic variation of chromatin patterns of cortical projection neurons across areas suggest that the epigenetic landscape of neurons also varies in parallel to laminar elaboration.

## Chromatin Patterns Differentiate as Cortical Neurons and Circuits Mature in Development

**FIGURE 6:**
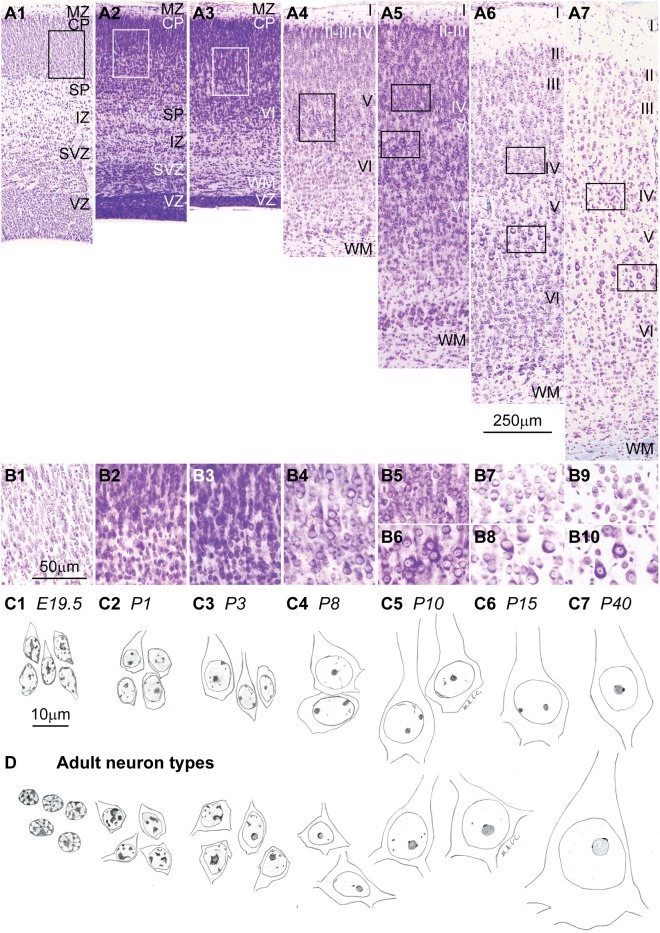
Cytodifferentiation of cortical neurons during development and in adulthood. **(A1–A7)** Nissl staining of area HL of the rat cerebral cortex during different embryonic (**A1**: E19.5) and postnatal (**A2**: P1; **A3**: P3; **A4**: P8; **A5**: P10; **A6**: P15; **A7**: P40) developmental stages show the expansion of the cortical plate (CP) and the formation of a eulaminate 6-layered cortex with the addition of new neurons, the progressive enlargement of their cytoplasm and nucleus, and the expansion of the neuropil. **(B1–B10)** High magnification of insets in **(A1–A7)** in the middle/deep cortical layers that show parallel changes in chromatin distribution patterns and size of the nucleus and cytoplasm associated with neuron maturation. Differentiation of neurons progresses from deep to superficial layers. **(C1–C7)** Sketches of developing layer V pyramidal neurons of the rat cerebral cortex from levels shown in **(B1–B4,B6,B8,B10)**. **(D)** Sketches of neuron types in the adult rhesus macaque; the left panel below **(C1)** depicts cerebellar granule cells; the panels below **(C2–C7)** depict progressively larger neurons of the cerebral cortex. In line with what LaVelle (1956), LaVelle and LaVelle (1970) suggested it appears that each stage in the development of nuclear architecture of the largest neurons (series in **C**) has a structural counterpart in the adult (series in **D**), in the form of some small neuron that never progressed beyond that stage of maturity. CP, cortical plate; IZ, intermediate zone; MZ, marginal zone; SP, subplate; SVZ, subventricular zone; VL, ventricular zone; WM, white matter. Roman numerals indicate cortical layers. Calibration bar in **(A6)** applies to **(A1–A7)**. Calibration bar in **(B1)** applies to **(B1–B10)**. Calibration bar in **(C1)** applies to **(C1–C7)** and **(D)**. (This figure is a reexamination of material from a gift of Dr. Alan Peters).

The morphological differentiation of cortical neurons follows a sequence described in classical studies using the Golgi stain. First, neuroblasts extend their axons as they migrate to the cortical plate. Once in their final position pyramidal neurons develop an apical dendrite, followed by the basal dendrites. Dendritic spines, intracortical axon collaterals of pyramidal neurons, and intracortical branches of white matter axons develop around the time of birth and postnatally, extending the distance between neurons (**Figure 6**). The neurofibrillary frame of the cytoskeleton develops postnatally (Ramón y Cajal, 1904/2002, 1929/1960; Marin-Padilla, 1970; Shoukimas and Hinds, 1978; Miller and Peters, 1981; Innocenti, 2011).

Classical studies with specific stains for DNA (Feulgen stain) and electron microscopy show that nuclear chromatin patterns in cortical neurons develop in parallel with morphological and cytoplasmic differentiation. The nuclei of migrating neuroblasts in the embryonic cerebral cortex of rodents have several clumps of heterochromatin (chromocenters), most of which are located under the nuclear envelope. As neuroblasts migrate and reach their position in the cortical plate, nucleolar substance gradually develops inside some chromocenters. Once in the cortical plate, nuclear heterochromatin disperses progressively and is redistributed around the developing nucleolus or nucleoli as Nissl substance appears in the cytoplasm (**Figure 6**; LaVelle and Windle, 1951; Meller et al., 1966; Caley and Maxwell, 1968; Butler and Caley, 1972). Postnatally, the number of cortical neurons with more than one nucleolus decreases (Marinesco, 1905; Ramón y Cajal, 1910; Buschmann and LaVelle, 1981; Miller and Peters, 1981; O’Kusky and Colonnier, 1982). The final size of the nucleolus and the number and thickness of perinucleolar heterochromatin clumps and grains depends on the size of the neuron body and the amount of Nissl substance produced (LaVelle and Windle, 1951; LaVelle, 1956; LaVelle and LaVelle, 1970; Buschmann and LaVelle, 1981; Pena et al., 2001). Interestingly, each stage in the development of the nuclear chromatin, the nucleolus and cytoplasmic Nissl substance has a counterpart in some type of adult neuron (LaVelle and LaVelle, 1970) as shown in **Figure 6**. In particular, the sequential changes during cytodifferentiation of large cortical neurons in embryonic and early postnatal development parallels the diversity of neuronal types seen in adults (**Figures 6C,D**).

The sequence of decision points that accompany neuron and glial cell differentiation during development is the result of changes in the transcriptome (Nowakowski et al., 2016, 2017; Fan et al., 2018; Zhong et al., 2018) that depend on modifications in the epigenetic landscape of nerve cells (Lister et al., 2013; Frank et al., 2015; Mo et al., 2015). The chromatin in the nucleus of cells during early embryonic development is mostly open and genes that are not transcribed are progressively silenced through the formation of tightly-arranged heterochromatin clumps. The more open the chromatin, the more multipotent the cells. Cell differentiation progresses through silencing of genes by methylation of DNA (Smith and Meissner, 2013), and post-translational modifications of histones like acetylation and methylation (Roidl and Hacker, 2014). At the end of embryonic development, terminally differentiated cells of a given type show a similar amount and distribution of heterochromatin, which differs across cell types (reviewed in Francastel et al., 2000; Melcer and Meshorer, 2010; Burton and Torres-Padilla, 2014). For example, all astrocytes in the adult central nervous system show the same nuclear chromatin pattern across brain regions [e.g., the white matter (Ling et al., 1973), cortex (García-Cabezas et al., 2016), and amygdala (Zikopoulos et al., 2016)]. In general, glial cell types show comparable chromatin patterns and nuclear size across species (**Figure 3**). Interestingly, neurons are an exception to this general pattern of chromatin differentiation and show different chromatin patterns depending on the size of their body and the type of connections they make (local circuit vs. longer-range interareal pathways; **Figures 1**, **2**).

**FIGURE 7:**
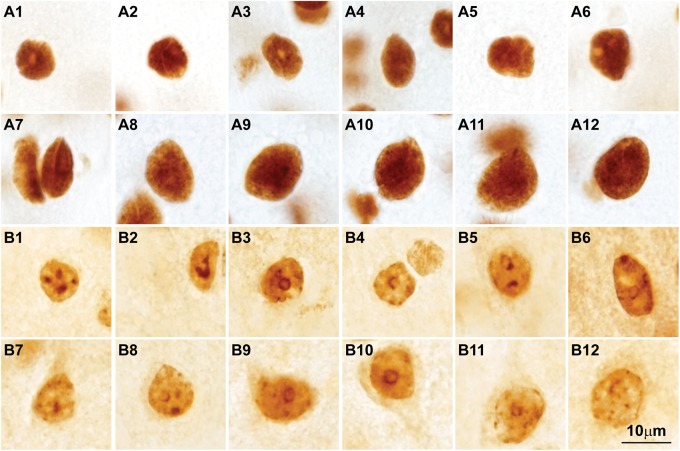
Post-translational histone modifications in the nuclei of cortical neurons. **(A1–A12)** Photomicrographs of cortical neurons in the primary visual cortex of the rhesus macaque labeled for H3K4met1-2-3 [Anti-mono/di/trimethyl-Histone H3 (Lys4), cat. #04-791, Millipore, Temecula, CA, United States], which shows a modification that facilitates gene expression; the nuclei of small and large neurons show intense H3K4met1-2-3 labeling with empty pockets that correspond to nucleoli and thick heterochromatin clumps. **(B1–B12)** Micrographs of cortical neurons in the primary visual cortex of the rhesus macaque labeled for H3K9met3 [Anti-trimethyl-Histone H3 (Lys9), cat. #07-442, Millipore], a repressive histone mark for gene expression; the labeling of H3K9met3 highlights heterochromatin grains and clumps with a distribution comparable to Nissl stained neurons (compare with **Figures 2B1–B15**). Calibration bar in **(B12)** applies to all panels.

## From Chromatin Patterns to Connections: Potential Disruption in Autism

The parallel development of chromatin patterns, neuron morphology, and pathways in the cerebral cortex summarized above suggests that specific epigenetic modifications in distinct types of cortical neurons underlie typical development of the cerebral cortex and its connections. This sequence of events in cortical neurons at the nuclear, cytological and connectional level could be disrupted in neurodevelopmental disorders like ASD. Specifically, changes in post-translational modifications of histone proteins have been reported in the brains of individuals with ASD, including a widespread increase of histone 3 (H3K4) methylation at gene promoters (Shulha et al., 2012a,b; Liu et al., 2016). Modification of histones during development may have an effect on neurogenesis and migration of neurons, which may be affected in ASD (Stoner et al., 2014; Marchetto et al., 2017), and in the pattern of gene expression and neuronal differentiation as shown for genes associated with the cell cycle and synapse formation (Pramparo et al., 2015; Liu et al., 2016). Findings of epigenetic dysregulation of post-translational histone modifications that lead to disruption of gene expression patterns vary among individuals, likely reflecting the heterogeneity of ASD. There is agreement on the prominent role of epigenetic dysregulation in neuronal synaptic dysfunction within the prefrontal cortex of individuals with ASD (Shulha et al., 2012a; Shen et al., 2014; Liu et al., 2016). In line with epigenetic alterations, studies at the cellular level have described changes in the cytoarchitecture and neurochemical features of neurons in the cortex of individuals with ASD (Bauman and Kemper, 2005; Casanova, 2007; Hutsler et al., 2007; Amaral et al., 2008; Schmitz and Rezaie, 2008; Simms et al., 2009; Avino and Hutsler, 2010; Hutsler and Zhang, 2010; Penzes et al., 2011; Schumann and Nordahl, 2011; Srivastava et al., 2012; Stoner et al., 2014; McKavanagh et al., 2015).

**Box 1| Principles, generalizations, and hypothesis.**

1. The nuclear pattern of chromatin and the size of the nucleolus of neurons is related to cell body size (Ramón y Cajal, 1896, Ramón y Cajal, 1910).
2. Each stage in the development of nuclear structure (nucleolus and heterochromatin) of the largest neurons is reflected in the adult through a structural counterpart (doppelganger) in the form of some small neuron that never progressed beyond that stage of maturity (LaVelle, 1956; LaVelle and LaVelle, 1970).
3. Terminally differentiated neurons show different nuclear chromatin patterns. By contrast, glial cells show comparable chromatin patterns by class (i.e., astrocytes, oligodendrocytes, and microglia).
4. We hypothesize that changes in chromatin pattern and epigenetic landscape, neuron size and connections vary systematically across cortical areas in parallel to the systematic variation of laminar structure of the cortex.
5. We hypothesize that analysis of epigenetic modifications in projection neurons in limbic areas of individuals with autism will reveal traces of developmental disruption.

Which are the cortical neurons most likely involved in ASD? Genetic studies have described a large variety of polymorphisms in individuals with ASD [see Samaco et al., 2005; Hogart et al., 2007; Weiss et al., 2009; Gilman et al., 2011; Hallmayer et al., 2011; Hussman et al., 2011; Shulha et al., 2012a; reviewed in Geschwind (2011)], which are typically found in open chromatin regions of cortical excitatory neurons (Lake et al., 2017; Fan et al., 2018), most of which likely are projection neurons. Our studies in the white matter of individuals with ASD show fewer thick and relatively more thin myelinated axons in white matter pathways below limbic areas of the anterior cingulate cortex, suggesting involvement of interareal pathways in ASD (Zikopoulos and Barbas, 2010; Zikopoulos et al., 2018). Taken together, this evidence points at projection neurons in the output layers (V-VI) of limbic cortical areas as the main neuron population involved in ASD. Alteration of cortical pathways in the white matter below limbic areas in ASD likely emerges from disrupted development. Could these changes in ASD be traced back to chromatin differentiation and epigenetic modifications during development of neurons and their connections? The answer to this question requires application of modern molecular and neuroanatomical techniques within a framework that takes into account the systematic variation of chromatin patterns, neuron morphology, laminar elaboration, and connections.

## Conclusion

Analysis of chromatin patterns across neuron types suggests that the observed variability reflects the structural and functional diversity of neurons and their connections, which entail successful execution of evolutionarily-conserved or -adapted developmental programs. Therefore, the relationship of nuclear chromatin patterns in cortical neurons with cell morphology and connections can be traced to development. Synthesis of the evidence summarized above and in **Box 1** leads to the testable hypothesis that the epigenetic landscape of neurons will vary systematically across cortical areas in parallel with laminar differentiation and connections. In the future, a wide array of techniques that allow sampling of thousands of cells in human postmortem brain tissue (Lister et al., 2013; Kozlenkov et al., 2016; Fullard et al., 2017, 2018; Lake et al., 2017) could be applied in the primate brain for cortical areas that have different laminar structure to test this hypothesis. Non-coding RNAs, a major epigenetic regulatory mechanism in neurons (Briggs et al., 2015), may also vary systematically across areas and networks. Differences in the epigenetic landscape in neurons across superficial and deep layers could be addressed with immunohistochemical studies of histone post-translational modifications as shown in **Figure 7**. Studies in human embryos on the development of the transcriptome (e.g., Nowakowski et al., 2017) and the epigenetic landscape (e.g., Lister et al., 2013) may reveal how systematic differences across areas emerge in development. These studies could provide the necessary framework to link the development of mature epigenetic states with neuron morphology and connections.

The same techniques could be applied in the brains of individuals with ASD to link epigenetic alterations with connectivity disruption. In particular, targeted analysis of epigenetic modifications, like DNA methylation and post-translational histone modification, in neurons in limbic areas of individuals with ASD may reveal traces of developmental disruption. According to our hypothesis, chromatin patterns and epigenetic marks in projection neurons of the main output layers of limbic cortices in individuals with ASD will differ compared to neurotypical subjects, reflecting developmental disruption.

## Ethics Statement

This study used archival, processed post-mortem brain tissue sections and was approved by the Institutional Review Board of Boston University, and by the Institutional Animal Care and Use Committee at Harvard Medical School and Boston University School of Medicine in accordance with NIH guidelines (DHEW Publication no. [NIH] 80-22, revised 1996, Office of Science and Health Reports, DRR/NIH, Bethesda, MD, United States).

## Author Contributions

MG-C, HB, and BZ conceived and wrote the manuscript. HB and BZ secured funding.

## Conflict of Interest Statement

The authors declare that the research was conducted in the absence of any commercial or financial relationships that could be construed as a potential conflict of interest.
